# Oxidative Damage and Autophagy in the Human Trabecular Meshwork as Related with Ageing

**DOI:** 10.1371/journal.pone.0098106

**Published:** 2014-06-19

**Authors:** Alessandra Pulliero, Anke Seydel, Anna Camoirano, Sergio Claudio Saccà, Marco Sandri, Alberto Izzotti

**Affiliations:** 1 Department of Health Sciences, University of Genoa, Genoa, Italy; 2 Dulbecco Telethon Institute, Venetian Institute of Molecular Medicine, Padua, Italy; 3 Department of Head/Neck Pathologies, St. Martino Hospital, Ophthalmology Unit, Genoa, Italy; 4 Department of Biomedical Sciences, University of Padua, Padua, Italy; 5 IRCCS AOU San Martino - IST, Genova, Italy; Oregon Health & Science University, United States of America

## Abstract

Autophagy is an intracellular lysosomal degradation process induced under stress conditions. Autophagy also plays a major role in ocular patho-physiology. Molecular aging does occur in the trabecular meshwork, the main regulator of aqueous humor outflow, and trabecular meshwork senescence is accompanied by increased oxidative stress. However, the role of autophagy in trabecular meshwork patho-physiology has not yet been examined in vivo in human ocular tissues. The purpose of the herein presented study is to evaluate autophagy occurrence in ex-vivo collected human trabecular meshwork specimens and to evaluate the relationship between autophagy, oxidative stress, and aging in this tissue. Fresh trabecular meshwork specimens were collected from 28 healthy corneal donors devoid of ocular pathologies and oxidative DNA damage, and LC3 and p62 protein expression analyzed. In a subset of 10 subjects, further to trabecular meshwork proteins, the amounts of cathepesin L and ubiquitin was analyzed by antibody microarray in aqueous humor. Obtained results demonstrate that autophagy activation, measured by LC3II/I ratio, is related with. oxidative damage occurrence during aging in human trabecular meshwork. The expression of autophagy marker p62 was lower in subjects older than 60 years as compared to younger subjects. These findings reflect the occurrence of an agedependent increase in the autophagy as occurring in the trabecular meshwork. Furthermore, we showed that aging promotes trabecular-meshwork senescence due to increased oxidative stress paralleled by autophagy increase. Indeed, both oxidative DNA damage and autophagy were more abundant in subjects older than 60 years. These findings shed new light on the role of oxidative damage and autophagy during trabecular-meshwork aging.

## Introduction

Autophagy is a highly conserved housekeeping pathway that plays a critical role in the removal of aged or damaged intracellular organelles and their delivery to lysosomes for degradation, [1], [2]. There are three major autophagic pathways that have been described, microautophagy, chaperone-mediated autophagy, and macroautophagy. Autophagy can be stimulated by a number of events including nutrient deprivation, exposure to pathogens, and oxidative stress. Microautophagy involves the direct phagocytosis of cytoplasmic elements and subsequent degradation of the elements in the lysosomal lumen. The second major type of autophagy is chaperone-mediated autophagy. Chaperone-mediated autophagy involves the delivery of proteins directly to the lysosome via chaperones such as Hsc70, [3]. The third and the best-characterized autophagic pathway is macroautophagy (hereafter referred to as autophagy). Autophagy follows a specific series of events, starting with initiation of formation of the phagophore. The phagophore elongates, engulfing a portion of cytoplasm containing the cargo to be degraded, and closes off to form the autophagosome. The final step of autophagy involves the fusion of the autophagosome with the lysosome to form the autophagolysosome, in which the gathered cargo is then degraded by lysosomal hydrolases. It has also been described the role of p53 tumor suppressor protein in regulating autophagy. In fact, depending on the p53 location, this protein exerts distinct and important function. Regarding this dual role, nuclear p53 acts as a transcription factor to stimulate both DRAM1 and Sestrin 2, which in turns switch on autophagy. In contrast, cytoplasmic p53 inhibits autophagy. In order to induce autophagy, p53 is finally degraded through proteosomes, [4].

Efficient autophagy or autophagocytosis is dependent on an equilibrium between the formation and elimination of autophagosomes; thus, a deficit in any part of this pathway will cause autophagic dysfunction. Autophagy plays a role in aging and age-related diseases [1], [2], [5]. Recent studies show that autophagy and changes in lysosomal activity are associated with both retinal aging and age-related macular degeneration, [6]. During autophagy, the cytosolic form of microtubule-associated protein 1A/1B- light chain 3 (LC3), LC3-I is processed and recruited to the phagophore where it undergoes site specific proteolysis and lipidation near the C terminus to form LC3-II [7]. Autophagy can be stimulated by a change of environmental conditions such as nutrient deprivation, various hormonal stimuli, and other factors. [7]. Recent studies have shed light on the importance of autophagy in both normal development, [8] tissue remodeling [7], and in pathological conditions [9]. Autophagy does not only serve to protect cells, but it may also contribute to cell damage. Induction of autophagy serves as an early stress response in axonal dystrophy and may participate in the remodeling of axon structures [10]. Autophagy also plays a major role in ocular patho-physiology. Morphological signs of autophagy have been described in the developing retina, participating in programmed cell death [11]. Molecular aging does occur in the trabecular meshwork (TM), the main regulator of aqueous humor outflow [12]. Oxidative damage in TM occur in primary open angle glaucoma [13] and is strictly related with intraocular pressure increase and visual field damage [14]. Indeed, dysfunction of the TM increases the resistance of the outflow pathway and induces an increase in intraocular pressure. The number of TM cells decreases with aging [15] the histopathologic findings of primary open-angle glaucoma tissue being similar to those of aged tissue [16], [17]. Reactive oxygen species (ROS) damage TM cells, induce apoptosis, and promote cellular aging in this tissue [18], [19]. Oxidative damage and the associated mitochondrial dysfunction may result in energy depletion, accumulation of cytotoxic mediators and cell death. TM cells of the human eye have been suggested as the proper model system for the study of the cellular aging [20]. It was reported that the number of TM cells decreases due to tissue damage induced by oxidative stress [21], [14] and senescence as occurring in glaucoma [22]. Oxidative stress responses, including metabolites redistribution alter the acetylation status of proteins, in human cells with mitochondrial dysfunction and in aging. On the other hand, autophagy and mitophagy eliminate defective mitochondria and serve as a scavenger and apoptosis defender of cells in response to oxidative stress during aging. These scenarios mediate the adaptation of cells to respond to aging and age-related disorders for survival. In the natural course of aging, the homeostasis in the network of oxidative stress responses is disturbed by a progressive increase in the intracellular level of the ROS generated by defective mitochondria [23].

Recent data demonstrate that the proteosome is inhibited in autophagy-deficient cells due to the accumulation of Nucleoporin 62 kDa (p62) [24]. p62 is a glycoprotein complex located in the nuclear membrane that is mainly implicated in trafficking proteins and mRNA between the nucleus and the cytoplasm [25]. Furthermore, p62 accumulates in autophagy-deficient mice suggesting a link between autophagy and p62 [26]. The reduced NF-κB activation observed in p62-deficient cells decrease ROS scavengers, which results in enhanced ROS levels and apoptosis. This mechanism explains the reduced carcinogenic potential of p62-deficient cells [27]. p62, a scaffold protein that binds ubiquitinated proteins and binds to LC3, recruiting the damaged mitochondria into the autophagosome, as well as stabilizing co-proteins such as Ambra1, [28]. In addition, p62 over-expression contributes to additional ROS production as part of an amplifying loop, thereby promoting genome instability [29]. Accordingly, there is plenty of evidence that autophagy is a major regulator in cell biology [30]. However, the role of autophagy in ocular patho-physiology has not yet been fully examined *in vivo* in human ocular tissues. In fact, there are in vitro studies of TM cells but not on human tissue samples. Only two studies analyzed oxidative stress in Porcine TM cells, as an in vitro model of aging, with increased lysosomal mass and content of autophagic vacuoles, decreased cathepsin L activity, and damaged mitochondria. [31], [32].

Our study is innovative as the first study of autophagy analyzed on samples of TM derived from human biopsies. The purpose of the herein presented study is to evaluate autophagy occurrence in ex-vivo collected human TM specimens and to evaluate the relationship between autophagy, oxidative stress, and aging in this tissue. These findings could serve to develop new preventive and therapeutic strategies to manage TM degenerative diseases such as glaucoma.

## Materials and Methods

### Trabecular Meshwork

We collected TM specimens from 28 healthy corneal donors devoid of ocular pathologies (kindly provided by the Melvin Jones Eyes Bank, Genoa, Italy). TM samples were taken from human scleral rings collected immediately after donor death as previously described, [33].

Cell viability was tested by MTT test and was always >90%. The study was approved by the Ethics Committee at the Genoa San Martino Hospital and University. The study ethical issues are also conform to the Declaration of Helsinki.

### Protein Lysates and Western Blot Analysis

**Figure 1:**
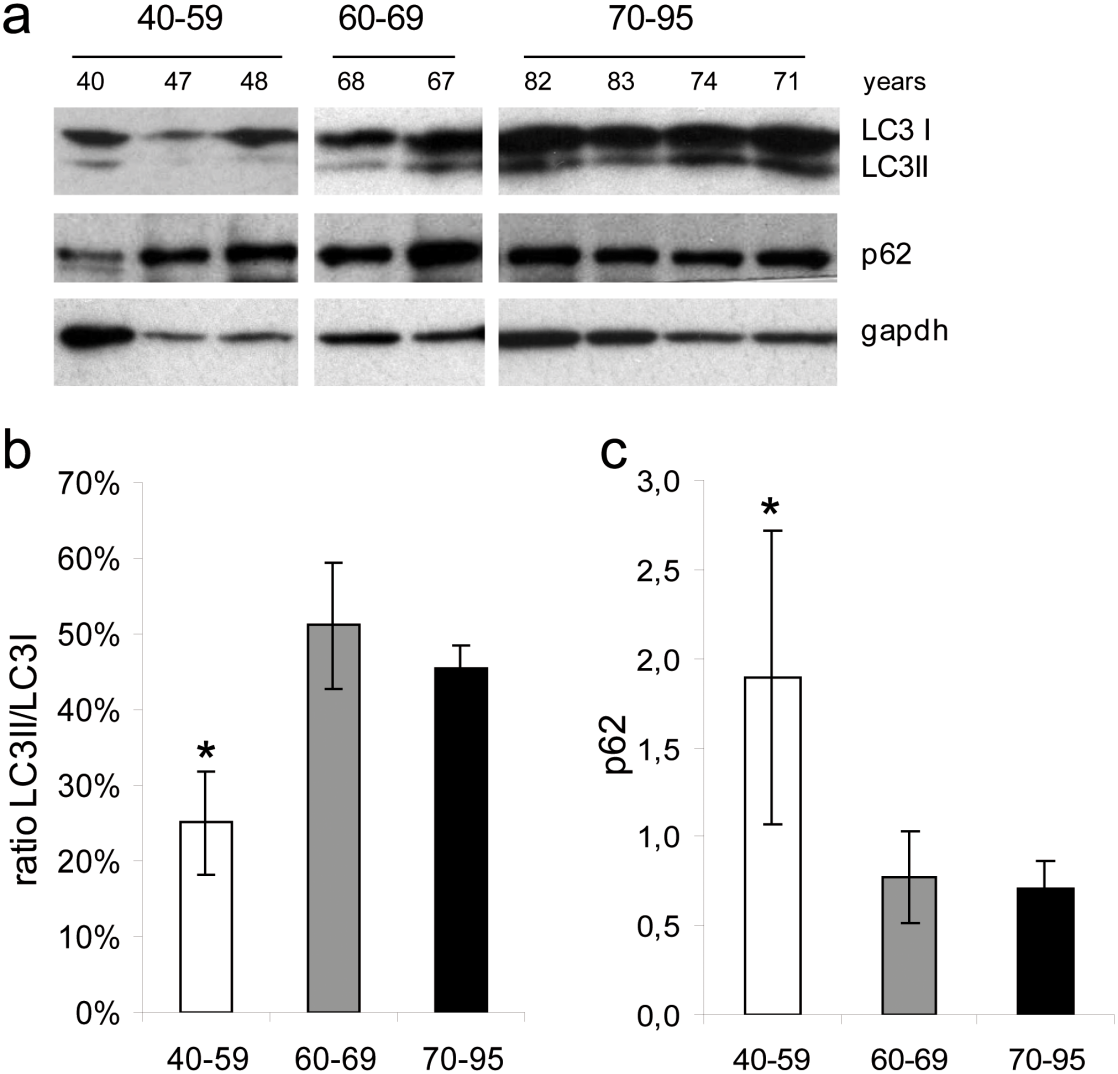
Western blot analysis of autophagy markers in human trabecular meshwork. (a) LC3II/I ratio was higher in elder (>60 years old) as compared to younger (<60 years old) subjects. (b) Conversely, p62 was lower in older subjects. These findings reflect the occurrence of an age-dependent blockage in autophagy as occurring in the TM. (c) Evaluation of LC3I, LC3II, and p62 proteins in the TM by Western blot. Protein amounts were compared at different age categories.

TM were taken from human sclerical rings without ocular pathologies. Tissue extracts were lysed in lysis buffer (50 mM Tris pH 7.5, 150 mM NaCl, 10 mM MgCl_2_, 1 mM EDTA, 10% glycerol, 5% SDS, 2% Triton, and protease inhibitor cocktails (Roche Diagnostics and Sigma Aldrich). After sonication on ice (30 pulses for 2 sec, at an amplitude of 30, Sonics Vibracell (Sonics & Materials, Newtown, CT USA) protein lysates were clarified by 10 min centrifugation, the supernatant was collected and protein content was quantified by BCA assay (Thermo Fisher Scientific Pierce Rockford, IL USA). Buffer containing 50 mM DTT (Invitrogen Carlsbad, Ca, USA) was added to standardized protein amounts, samples heated at 70° for 10 min., and proteins separated on NuPAGE Novex 4–20% Tris-Glycine gels (Invitrogen Carlsbad, Ca, USA) and then transferred to PVDF membranes (Biorad Hercules, CA USA). Subsequently, membranes were blocked with 5% non-fat dry milk (Biorad Hercules, CA USA) in PBS-T and incubated overnight with specific primary antibodies: LC3 (2775S, Cell Signaling Tecnology Danvers MA USA), p62 (POD67, Sigma-Aldrich Saint Louis Missouri USA) and GAPDH (8245, Abcam Cambridge Science Park England). Antibodies used and their diluitions are reported in **Table 1**. Accordingly, the membranes were then incubated with secondary anti-rabbit or anti-mouse antibodies conjugated to horseradish peroxidase. Bands were detected by incubating the membranes with ECL (Thermo Fisher Scientific Pierce Rockford, IL USA). The differences in expression levels of LC3-II and LC3-I (**Figure 1**) are determined by multiple scans of blots to ensure a maximum and minimum response range for the measured areas, and the integrated areas of the bands are calculated using the software Image J [34]. Appropriate background subtraction and normalization of the data to GAPDH is done for each lane before calculating the LC3-II to LC3-I ratio.

**Table 1:** Primary antibodies used for western blots protein analysis in trabecular meshwork.

target protein	Antibody dilution	Company
GAPDH	1∶2000 polyclonal anti-mouse	Abcam
LC3I/LC3II	1∶1000 polyclonal anti-rabbit	Cell Signaling
p62	1∶1000 polyclonal anti-rabbit	Sigma-Aldrich

Western blot analyses were replicated in 4 independent experiments.

Statistical significance of difference in protein amounts between sample groups was calculated by t test for unpaired data.

### 8-oxo-dG

Oxidative DNA damage was determined by quantifying 8-hydroxy-2-deoxyguanosine (8-oxo-dG), the most important and abundant indicator of oxidative lesions of DNA in chronic degenerative diseases. 8-oxo-dG has also been demonstrated to be present at high levels in the TM of glaucomatous eyes [13]. This molecule results from the interaction between the hydroxyl radical OH and the C2 of guanine, resulting in a hydroxylated guanine that, if unrepaired by specific glycosylases, may cause G3A transversions and formation of apurinic sites. The analytical method used for 8-oxo-dG detection was ^32^P postlabeling and thin-layer chromatography. DNA (1 µg) was depolymerized to 3-monophosphate nucleotides by incubation with micrococcal nuclease (0.14 U/µg DNA) and spleen phosphodiesterase (1 mU/µg DNA) at 37°C for 3.5 hours. Unmodified dGp nucleotides were selectively removed by incubation with 80% vol/vol trifluoroacetic acid (30 µl) for 10 minutes at room temperature. Samples were dried by vacuum centrifugation, and 3′-phosphate-8-oxo-dG was labeled by incubation with T4 plasmid polynucleotide kinase (8U) dissolved in 200 mM bicine, 100 mM DTT, and 10 mM spermidine. Reaction in the presence of AT-gamma-^32^P (64 µCi, specific activity 750 Ci/mmol; ICN, Irvine, CA, USA) was conducted at 24°C for 40 minutes. The mixture underwent nuclease P1 digestion (2.7 U at 37°C for 60 minutes) to selectively separate ^32^P from normal nucleotides. ^32^P-labeled 8-oxo-dG was purified from the reaction mixture by monodirectional thin-layer chromatography on an 18×3-cm cellulose sheet coated with the anion exchanger polyethylenimine (Macherey and Nagel, Düren, Germany). The chromatographic development was performed in unbuffered 1.5 M formic acid. Under these conditions, ^32^P-labeled 8-oxo-dG slowly migrates to the central part of the chromatographic area, whereas normal nucleotides accumulated on the upper edge of the chromatographic sheet that was cut away. ^32^P-labeled 8-oxo-dG was identified by electronic autoradiography and quantified by measuring the emitted radiation using a ^32^P imager (Instant Inmager; Packard, Meriden, CT, USA). Positive reference standards were obtained by incubating calf thymus DNA with 1 mM CuSO4 and 50 mM H_2_O_2_ or using an authentic 8-oxo-dG reference standard (National Cancer Institute Chemical Carcinogen Reference Standard Repository; Midwest Research Institute, Kansas City, MO). DNA-free samples were used as a negative control.

### Antibody Microarray

Due to the strict relationship between TM and aqueous humor proteome composition [35], [36], the amount of autophagy related proteins was evaluated in aqueous humor. The amount of cathepesin L and ubiquitin was tested in aqueous humor of 10 subjects for which both TM and aqueous humor were available. Indeed, cathepsin L and ubiquitin play a major role in autophagy. Cathepsin L degrades lysosomal membrane components, GABARAP-II and LC3-II [37] and ubiquitin proteasome system is a major intracellular protein degradation pathway [23]. Due to the small amount of protein in the aqueous humour samples collected, standard Western blotting methods could not be used to analyze the expression of these proteins in the available samples. Accordingly, we decided to use the antibody microarray method [38]. The analysis was conducted using Clontech Ab Microarray TM 500 s (Clontech, CA, USA), including both cathepsin L and ubiquitin. Each aqueous humor sample (150 µl) were diluted in Extraction/Labeling Buffer (Clontech, CA, USA) and labeled via a 2-hour incubation at 4°C with either Cy3 or Cy5. Cy3 and Cy5 were formulated as monofunctional protein-reactive dyes binding amino groups (GE Healthcare, UK). The labeled proteins were purified via elution in 100 µl of 1x Desalting Buffer in Protein Desalting Spin Columns (Pierce Biotechnology, Il, USA). Purified and labeled proteins were quantified using the BCA assay with a NanoDrop ND-1000 (a nano-spectrophotometer from Nanodrop Technologies, DE, USA) and then hybridized onto glass antibody microarrays (Explorer Antibody Microarray, Full Moon BioSystems Inc.). The hybridization was performed at room temperature for 40 min with continuous shaking. The slides were washed in 3 different washing buffers (Clontech, CA, USA) for 5 min each at room temperature with gentle rocking, dried by centrifugation, and analyzed by laser scanning and fluorescence detection (ScanArray Lite, Packard Bioscience). Signal quantification was performed by the QuantArray software (GSI Lumonics), which subtracted the local background fluorescence from the signal intensities. The used antibody microarray was then spotted with two replicate probes. Protein concentrations were expressed as fluorescence intensity units (FUs) that were related to the intensity of the fluorescent signal that was detected for each spot that had been coated with the corresponding antibody. An internal standard of albumin was used in each sample to perform a quantitative standardization of the obtained results.

Proteome expression were analyzed using version 7.3 of the Genespring software package (Agilent Technologies, Santa Clara, CA, USA). Raw data from which the backgrounds had been subtracted were log transformed and normalized both per chip and per protein by median centering.

## Results

### Western Blot Analysis

#### LC3 and p62 protein expression in trabecular meshwork

Protein expression of LC3I, LC3II, and p62 in the TM were analysed by Western blot (**Figure. 1**). The protein expression at different ages (**Figure. 1a**) were compared among three different categories (40–59, 60–69, 70–95 years). The ratio LC3II/I was significantly higher in 70–95 years old as compared to 40–59 years old subjects. The ratio in the three age groups was 25%, 50%, and 45%, respectively from young to old (**Figure. 1b**). The p62 expression higher in 40–59 than in 60–69 and 70–95 years groups (P<0.05) (**Figure. 1c**).

#### 8-oxo-dG

**Figure 2:**
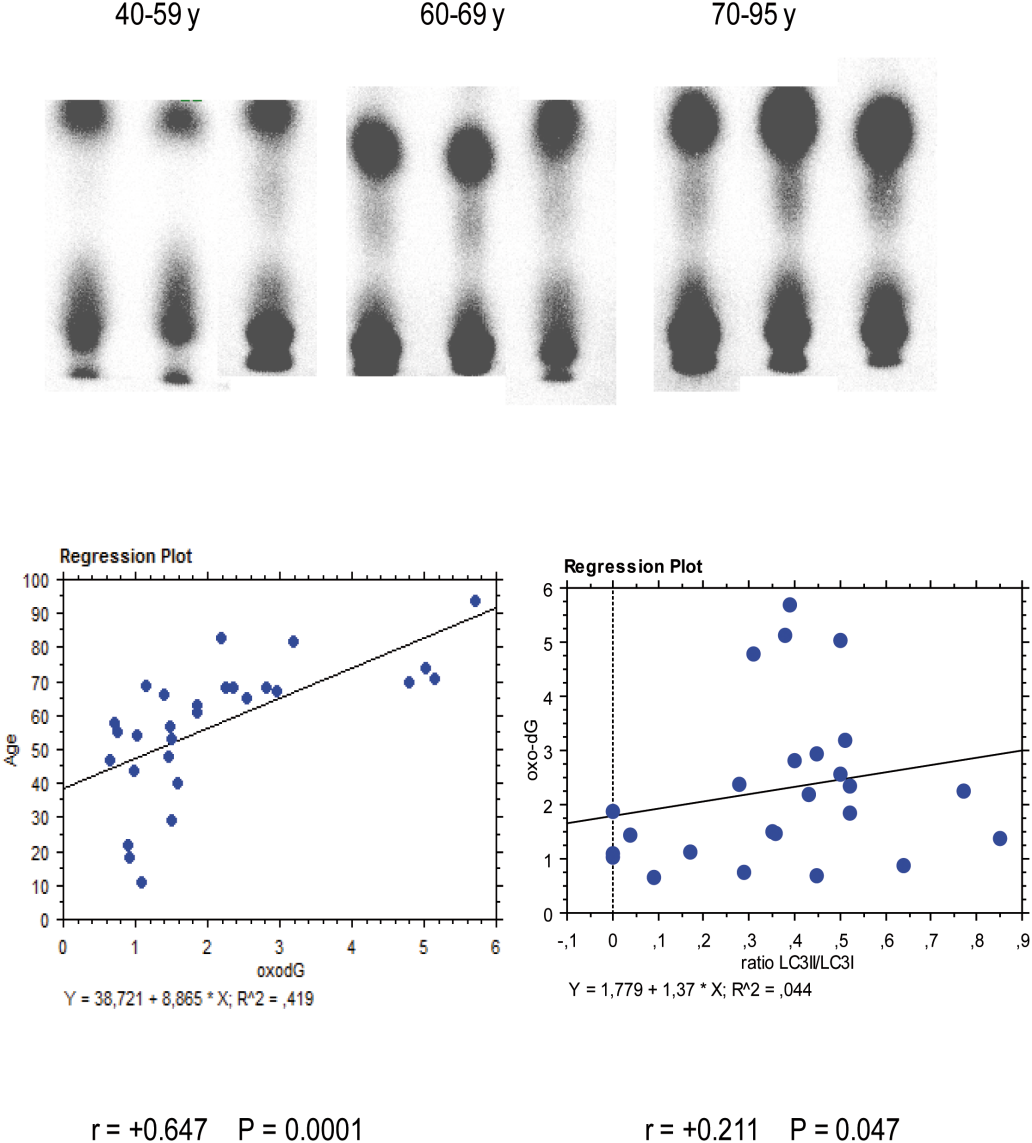
Examples of 8-oxo-dG radioactive spots as detected by 32P-postlabelling (left panel). For each sample the lower spot is the origin of chromatography, the upper spot 8-oxo-dG. Linear regression analysis (right panel) indicates that oxidative DNA damage in TM (horizontal axis) was significantly (r = +0.647, P<0.0001) correlated with age (vertical axis). A significant relationship between DNA oxidative damage and autophagy activation as measured by LC3 II/I ratio was detected (r = +0.211, P = 0.047) (lower panel).

Examples of 8-oxo-dG radioactive spots detected by ^32^P-postlabeling are reported in **Figure. 2** **.** A significant relationship between age and 8-oxo-dG amount was observed. Indeed 8-oxo-dG was 1.13±0.34 8-oxo-dG/10e5 nucleotides (mean±SD) in young (40–59 years), 1.31±0.39 in middle aged (60–69 years), and 3.13±1.45 in old (70–95 years) (P<0.01 ANOVA). Linear regression analysis indicates that oxidative DNA damage was significantly correlated with age (y (age) = 38.72+8.87×(8-oxo-dG); r = 0.647; P<0.0001. (**Figure. 2**). A relationship between DNA oxidative damage and autophagy activation as measured by LC3 II/I ratio was detected (r = +0.211, P = 0.047) (**Figure. 2**, lower panel).

#### Antibody microarray

**Figure 3:**
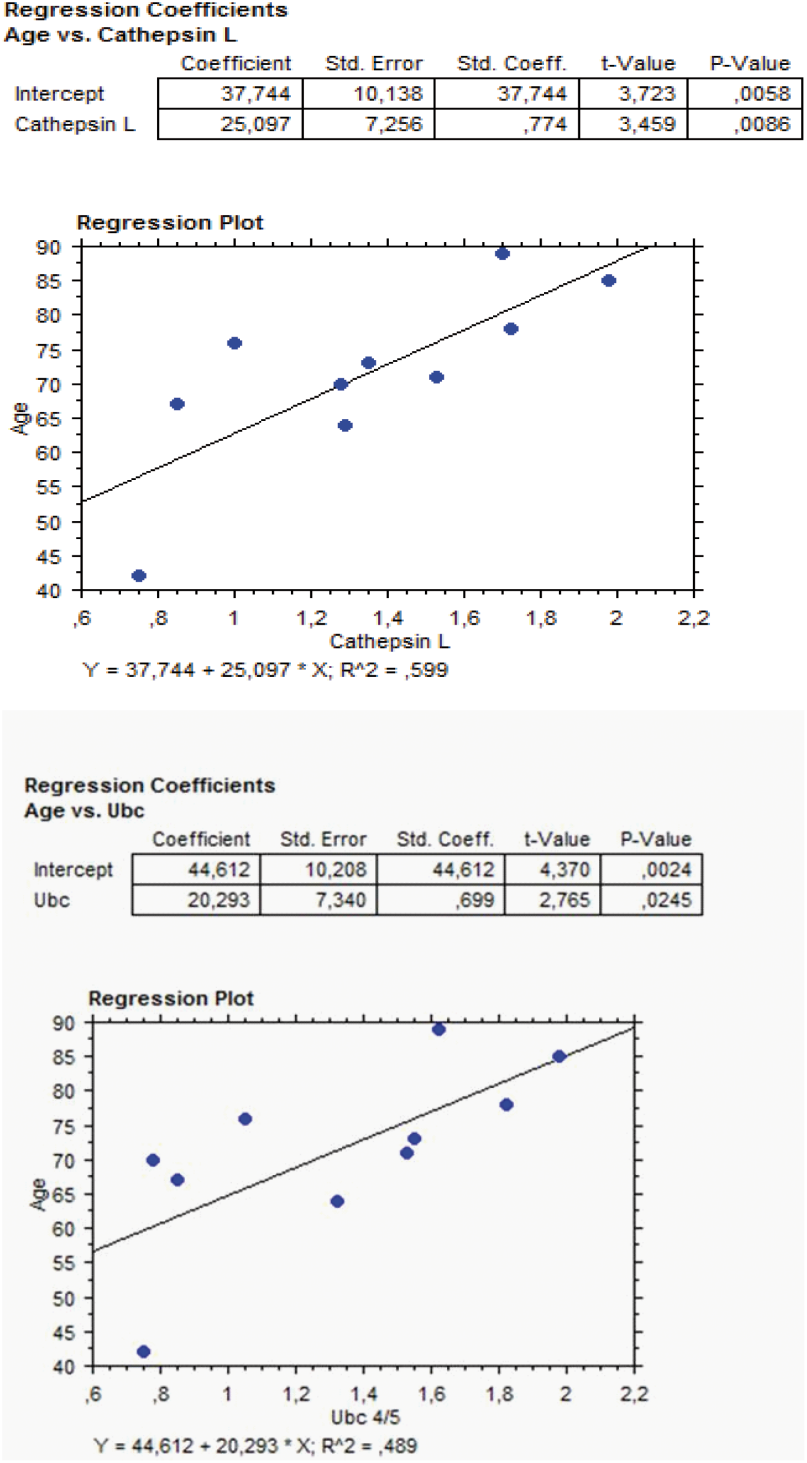
Regression analysis plots of the significant relationship between Age (vertical axis) and amounts of Cathepsin L (horizontal axis, left panel, r = +0.774, P = 0.0086) and Ubiquitin (horizontal axis, right panel, r = +0.699, P = 0.0245) as evaluated in aqueous humor by antibody microarray. These two proteins are directly involved in autophagy.

Ten out of 28 examined subjects underwent analysis of cathepsin L and ubiquitin proteins in aqueous humor as performed by antibody microarray. The amounts of these proteins were significantly related with age, as evaluated by linear regression analysis (**Figure. 3**). The relationships were as follows: (A) cathepsin L and age: (y (age) = 37.74+25.09×(cathepsin L); r = 0.774; P = 0.0086; (B) ubiquitin and age: (y (age) = 44.61+20.29×(ubiquitin); r = 0.699; P = 0.0245.

## Discussion

Chronic oxidative stress, inflammation, and accumulation of protein-rich deposits occur both in TM endothelium and in retinal pigment epithelium heralding the onset of age-related degeneration. It is known that cell survival decreases with age due to apoptosis activation [39]. Furthermore, as shown in this study, aging also promotes in TM the increase of both oxidative damage and autophagy. The increase of autophagy is consequent to the age-related accumulation of oxidative damage, as highlighted in subjects older than 60 years. Four specimens with high oxo-dG strongly influenced the correlation between ixudativedamge and autophagy. Because post mortem intervals were accurately standardize among samples, it is likely that high levels of oxidative signaling are required to increase autophagy as a protective mechanism against TM degeneration.

An autophagy receptor is the ubiquitin recognition protein p62, [40]. This protein has been shown *in vitro* to bind both ubiquitin and LC3, a protein localized in the pre-autophagosomal and autophagosomal membranes. This interaction is the mechanism by which p62 brings selected proteins to the autophagosome system for degradation [41]. Accordingly, p62 amount is inversely related with autophagy activation [9] being decreased through autophagy-mediated degradation [42]. p62 is a connecting link between autophagy and proteasome mediated proteolysis, and its expression is altered by exposure to oxidative stress [43]. The p62 protein serves as a link between LC3 and ubiquitinated substrates, p62 being incorporated into autophagosome and driving autolysosome degradation [25].

Herein presented results, provide evidence that p62 is lower in subjects older than 60 years as compared to younger subjects. These findings reflect the occurrence of an age-dependent increase in autophagy as occurring in the TM. The increased autophagy events during TM aging is supported by the increase in the LC3 II/I ratio we detected in older subjects. LC3 II/I ratio is one of the indicators of autophagy activation [37]. LC3 assists autophagosome formation enhancing membrane fusion. When autophagy is activated, the soluble cytosolic LC3I bind lipid phosphatidyl ethanolamine being transformed into the LC3II lipidated form anchoring the autophagosomal membranes. LC3-II remains associated with autophagosomal membrane until its fusion with the lysosome, thus serving as a bona fide marker of autophagy activation [44]. The development of molecular and imaging tools to follow autophagosome formation has greatly improved the characterization of autophagy in normal and atrophying muscles [45], [46].

Autophagy is typically activated in cells undergoing oxidative stress [47] and mitochondrial damage [23]. Presented results indicate that LC3II/I ratio and oxidative damage are tightly related during TM aging.

Our previous studies showed that mitochondrial DNA deletion is dramatically increased in TM of patients with primary open angle glaucoma versus controls. This finding was paralleled by a decrease in the number of mitochondria per cell and by cell loss [48]. In the aging process, accumulation of mitochondria DNA mutations, impairment of oxidative phosphorylation as well as an imbalance in the expression of antioxidant enzymes result in ROS overproduction. Autophagy and mitophagy eliminate defective mitochondria and serve as a scavenger and apoptosis defender of cells in response to oxidative stress during aging. In the natural course of aging, the homeostasis of oxidative stress responses is disturbed by a progressive increase of ROS generated by defective mitochondria, [23].

These mechanisms play a major role in ocular patho-physiology. In lens epithelium, autophagic vesicles containing mitochondria are produced during the early stages of lens cell differentiation [49]. TM is located at the angle of the anterior chamber of the eye and contains endothelium-lined spaces through which the aqueous humour passes to the Schlemm’s canal. TM possess a remarkable ability to modify its permeability by changing cell shape and tissue morphology by contracting its cells, and is one of the tissues involved in maintaining appropriate levels of IOP. Elevated IOP occurs when the amount of aqueous humor entering the anterior chamber of the eye cannot exit through the TM conventional outflow pathway [50]. Resistance to aqueous humor outflow increases with aging, although the molecular mechanisms responsible are not clear yet, [51]. Acceleration in the production of ROS causes oxidative damage to the TM with aging and contribute to the observed loss in TM tissue functionality in ocular hypertension and in primary open angle glaucoma (POAG) [12], [13], [52].

Our results provide evidence that a relationship between autophagy, oxidative damage, and aging occur in TM also reflecting in aqueous humor composition. Cathepsin L and ubiquitin expression protein are directly involved in the autophagosome function. Accordingly, their finding in aqueous humor and their significant relationship with aging indicate that autophagy is an age-related event occurring in ocular anterior chamber tissues. Our previous studies demonstrated that aqueous humor proteins alteration reflect proteome changes occurring in TM, as demonstrated by analysing samples collected from glaucoma patients [35], [36]. Indeed, chronic exposure in vitro of TM cells to oxidative stress, causes profound changes in the lysosomal system, including increased lysosomal mass and content of autophagic vacuoles, accumulation of intralysosomal oxidized material and damaged mitochondria, as well as decreased cathepsin L activity [31].

Proteolytic activation of cathepsins can be facilitated either by autocatalytic activation at acidic pH, by activation by other proteases, or both. Since lysosomal proteases are optimally active in the acidic pH, such an increase in lysosomal pH could certainly explain the overall decrease in cathepsin activities in TM cultures, either by directly affecting the autocatalytic activation or indirectly by interfering with the activation of other proteases required for proteolytic cleavage, [52].

Aging results from the gradual decline in cellular repair and housekeeping mechanisms, which leads to an accumulation of damaged cellular constituents and ultimately to the degeneration of tissues and organs. Autophagy promotes cell maintenance by removing accumulated toxic material and by using recycled components as an alternative nutrient resource [53]. This suggests that autophagy favors longevity because an organism can recover more quickly from stress-induced cellular damage. Our results provide evidence that, under physiological situation, autophagy increases with age in human TM. In future studies it will be of interest to evaluate if this process is impaired under pathological situations affecting TM, such as glaucoma.
